# Rapid Improvement in Consciousness After Low-Dose Ketamine in a Patient With Acute Confusion: A Case Report

**DOI:** 10.7759/cureus.98635

**Published:** 2025-12-07

**Authors:** Waleed Khalid Khalafallah Khalid, Hind Abdelazim Mirghani Ibrahim, Ibtisam Ahmed Abdullah Al Hoqani

**Affiliations:** 1 Emergency Medicine, Royal College of Emergency Medicine, London, GBR; 2 Emergency Medicine, Khoula Hospital, Muscat, OMN; 3 Emergency Medicine, Ahfad University For Women, Omdurman, SDN

**Keywords:** acute confusion, delirium, emergency medicine, gcs recovery, glasgow coma scale, intravenous ketamine, ketamine, procedural sedation, rapid arousal, urinary tract infection

## Abstract

Ketamine, widely used for procedural sedation, may possess underrecognized neuroprotective and cognitive-modulating effects. We report the case of a 66-year-old female patient who presented with acute confusion (Glasgow Coma Scale (GCS) 12/15; E3V4M5), likely secondary to a urinary tract infection. Remarkably, she regained full consciousness (GCS 15/15; E4V5M6) within 45 minutes of receiving 40 mg of intravenous ketamine for nasogastric tube insertion. No other sedatives or interventions were administered during this period. This observation raises the possibility that ketamine may play a role in rapidly reversing acute confusion and enhancing arousal in select clinical scenarios. While anecdotal, such findings align with emerging literature and warrant further exploration of ketamine’s neurocognitive effects beyond its established indications.

## Introduction

Ketamine, an N-methyl-D-aspartate (NMDA) receptor antagonist, is widely recognized as a versatile drug in emergency medicine. It is commonly used for sedation, analgesia, asthma exacerbation management, seizure control, rapid sequence intubation (RSI) in hypotensive patients, awake intubation, and cerebroprotection in head trauma. Ketamine’s diverse therapeutic applications have made it a favorite among emergency physicians. Traditionally, ketamine has been widely used in emergency care for procedural sedation, analgesia, reduction of dislocations, burn dressing changes, and as an adjunct in the management of seizures and severe asthma. These diverse effects are mediated through NMDA receptor antagonism. In addition to these established uses, emerging literature highlights its potential in enhancing cognition, reducing delirium, improving levels of consciousness, and treating major depressive disorder resistant to standard therapy [1-5]. Ketamine has also been studied for its potential to reduce postoperative delirium and cognitive decline, especially in elderly surgical patients [6,7]. This case illustrates ketamine’s surprising neuroactivating effect in the context of acute confusion.

## Case presentation

A 66-year-old female patient with end-stage renal disease on regular haemodialysis, breast cancer on hormonal therapy, long-standing hypertension, and a history of recurrent urinary tract infections (UTIs) presented to the Emergency Department (ED) with acute confusion characterised by fluctuating attention, severe disorientation to time, place, and person, impaired comprehension, and marked agitation. Her symptoms had been gradually progressive over approximately 26 hours. The confusion had progressed gradually. On arrival, her Glasgow Coma Scale (GCS) was 12/15 (E3V4M5).

Vital signs showed a blood pressure (BP) of 195/85 mmHg, heart rate (HR) 81 beats per minute, respiratory rate (RR) 18 breaths per minute, temperature 38.1°C, and oxygen saturation (SpO₂) of 96% on room air. Neurological examination showed marked agitation and disorientation to time, place, and person. Cranial nerves were grossly intact. Muscle tone was normal, but power could not be reliably assessed due to poor cooperation. Deep tendon reflexes were normal, and there were no signs of meningeal irritation or focal neurological deficits. Chest auscultation was clear, and heart sounds were normal.

**Figure 1:**
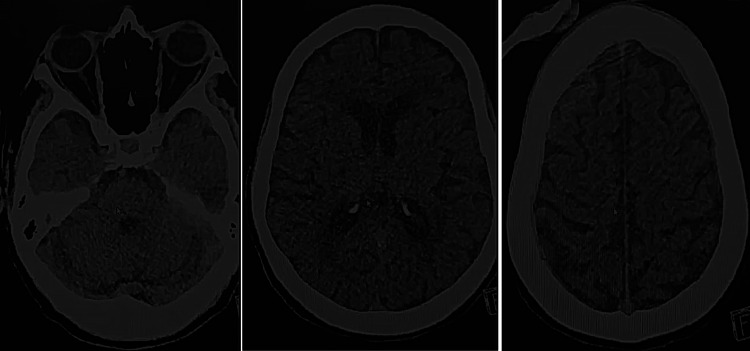
Non-contrast CT Head (Axial Slices) Three axial non-contrast CT images demonstrating no acute intracranial pathology.

Urinalysis revealed leukocytes (+), and urine microscopy showed 20 WBCs per cumm, consistent with an acute UTI, and her fever (38.1°C) supported this impression [8]. Cerebrospinal fluid (CSF) analysis was clear, with normal glucose and no organisms on Gram stain. A non-contrast CT of the head showed no acute intracranial pathology (Figure 1). The CT findings support that the patient’s confusion was not due to an acute structural brain lesion.

The official radiology report for the CT scan (Figure 1) indicated no acute intracranial pathology. Although the study was mildly degraded by motion artefact, the radiologist noted only age-related atrophic changes, chronic small-vessel disease, and a chronic right basal ganglia lacunar infarct. No hemorrhage, mass effect, midline shift, or herniation was present. A small right intraventricular isodense lesion was described without associated hydrocephalus or ventriculomegaly. Overall, there was no CT evidence of an acute intracranial insult.

Initial laboratory investigations included complete blood count, renal function tests, liver panel, electrolytes, C-reactive protein (CRP), and ammonia. These are detailed in Table 1. Findings were consistent with her baseline chronic renal impairment, with no significant new derangements. A diagnosis of acute confusion secondary to UTI was considered.

**Table 1:** Laboratory Investigations GFR: glomerular filtration rate; eGFR: estimated glomerular filtration rate; MDRD: Modification of Diet in Renal Disease

Parameter	Patient Value	Reference Range
Haemoglobin in Blood	11.7 g/dL	11-14.5
White Blood Cells in Blood	6.00x10^3^/uL	2.4 - 9.5
Platelet count in Blood	140x10^3^/uL	150 - 450
Red Blood cells	3.38x10^6^/uL	4.1 - 5.4
Haematocrit of Blood	34.5 %	34 - 43
Mean Cell Volume	102.1 fL	78-95
Mean Cell Hemoglobin	34.6 pg	26 - 33
RBC distribution width	13.1 %	11.5 - 16.5
Neutrophils # in Blood	3.90x10^3^/uL	1-4.8
Lymphocytes # in Blood	1.16x10^3^/uL	1.2 - 3.8
Eosinophils # in Blood	0.22x10^3^/uL	0 -. 5
Monocytes # in Blood	0.66x10^3^/uL	.1 - 1.3
Basophils # in Blood	0.06x10^9^/L	0 - .2
Mean Cell Hb Conc	33.9 g/dL	31 - 35
Mean Platelet Volume in Blood	11.3 fL	7 - 10.5
Lactate in Serum/Plasma	1.40 mmol/L	.5 - 2.2
C-Reactive Protein in Serum	1.30 mg/L	0 - 5
Aerobic Blood Culture	No bacterial growth	
Anaerobic Blood Culture	No bacterial growth	
Urine Culture	No bacterial growth	
White Cells in Urine	20 cells/cumm	0 – 5 cells/μL
Red Cells in Urine	24 cells/cumm	0 – 3 cells/μL
Epithelial cells in Urine	4 /uL	0 – 5 cells/μL
Casts in Urine	NIL/HPF	None seen
Crystals in Urine	NIL	None seen
Bacteria in Urine	NIL	None seen
Yeast in Urine	NIL	None seen
Urine Analysis		
Leukocytes (White Cells)	+	Negative
Erythrocytes (Red Cells)	Trace	Negative
Glucose in Urine by Test	NIL	Negative
Bilirubin in Urine	NIL	Negative
pH of Urine	7.5	4.5 – 8.0
Ketones in Urine	Negative	Negative
Specific Gravity of Urine	1.010	1.005 – 1.030
Nitrite in Urine	NIL	Negative
Protein in Urine	+++ g/L	Negative
Urobilinogen in Urine	Negative	Negative
Cell count & Differential in Body Fluid (CSF), Panel		
White Blood Cells CSF	3/uL	0 – 5 cells/μL
Polymorphonuclear cells % CSF	33 %	0 – 6%
Mononuclear cells % in CSF	67 %	94 – 100%
Red Blood Cells in CSF	1x10^3^/UL	0 – 10 cells/μL
Gram-negative Bacilli	Not seen	Not seen
Gram-negative Cocci	Not seen	Not seen
Gram-positive Cocci	Not seen	Not seen
Gram-positive Bacilli	Not seen	Not seen
Yeast Cells	Not seen	Not seen
Site/Type of Specimen	LP CSF	
Appearance of Specimen	Clear	Clear
Gram Film	No organisms seen	
CSF Culture	No bacterial growth	
Protein in CSF	70.01 mg/dL	15 - 45
Glucose in CSF	2.92 mmol/L	2.22 - 3.89
Ammonia in Plasma	16.50 umol/L	11 - 51
Urea in Serum/Plasma	13.9 mmol/L	2.7 - 8.07
Creatinine in Serum/Plasma	587.84 umol/L	44 - 80
Sodium in Serum/Plasma	133.37 mmol/L	136 - 145
Potassium in Serum/Plasma	4.26 mmol/L	3.5 - 5.1
Chloride in Serum/Plasma	95.95 mmol/L	98 - 107
eGFR.MDRD	7 mL/minute/1.73 m^2^	90 - 120
Bilirubin Total in Serum	13.18 umol/L	3 - 21
Protein Total in Serum/PI	65.52 g/L	64 - 80
Alanine Transaminase in serum	7.09 U/L	0 - 33
Alkaline Phosphatase in serum	55.70 U/L	35 - 104
Albumin in Serum/Plasma	40.40 g/L	35 - 52

**Figure 2:**
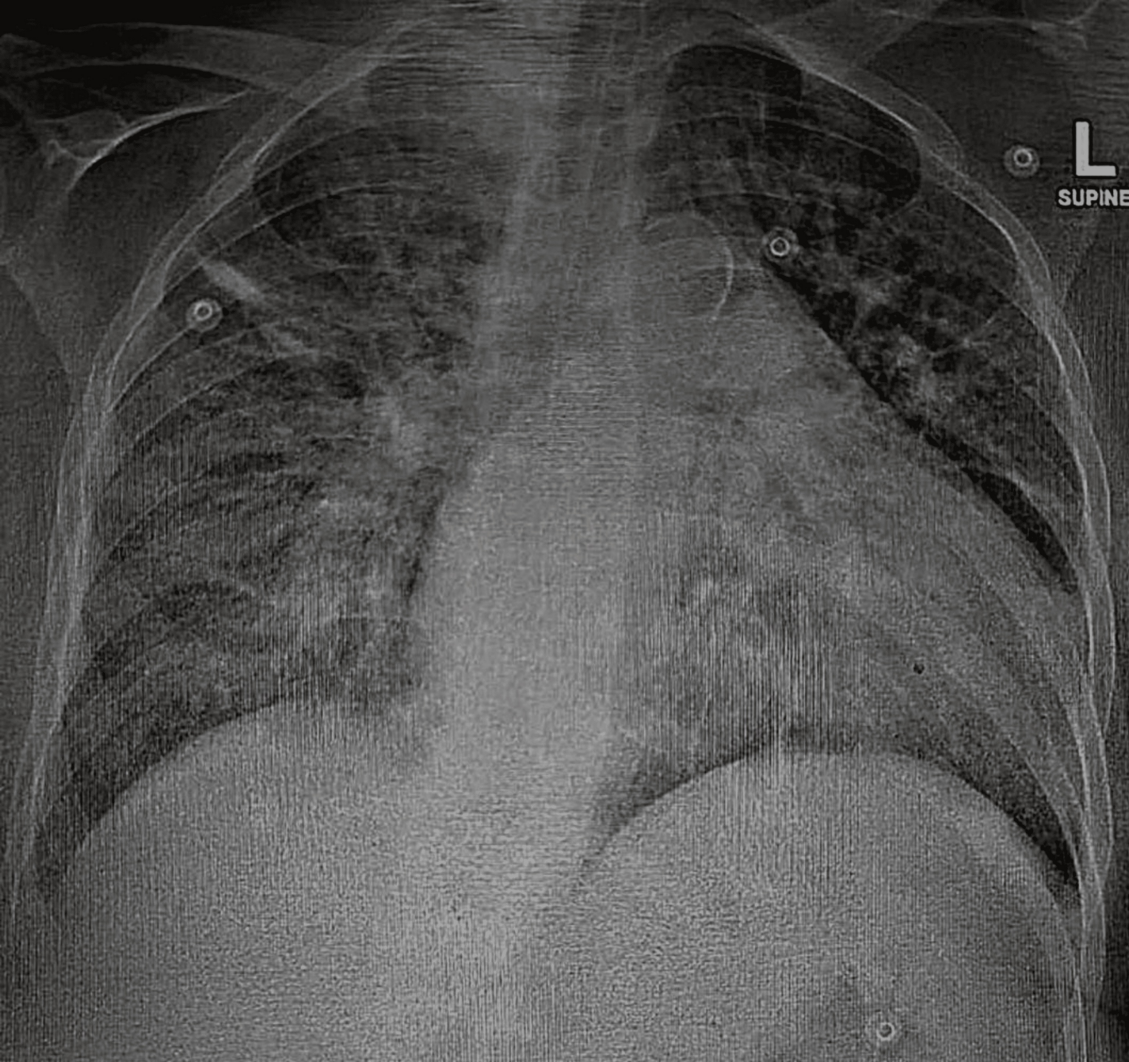
Chest X-ray Confirming nasogastric tube position with no acute pulmonary findings.

Due to significant agitation, poor cooperation, and inability to take oral medications for over 24 hours, intravenous ketamine, 40 mg (0.5 mg/kg based on an estimated body weight of 80 kg), was administered for procedural sedation to facilitate nasogastric tube (NGT) insertion. Over the next 45 minutes, three NGT insertion attempts were made. During this period, the patient became calm enough to allow the NGT attempts. A chest X-ray was subsequently performed to confirm NGT placement and showed no acute pulmonary abnormalities (Figure 2). This was clinically relevant as it excluded pulmonary infection, aspiration, or respiratory pathology as contributors to her altered mental status. No other medications were administered during this period.

Remarkably, 45 minutes after ketamine administration, the patient regained full consciousness (GCS 15/15; E4V5M6). She became fully oriented, cooperative, and interacted appropriately. Post-recovery vital signs were BP 197/90 mmHg, HR 82 bpm, RR 18 breaths/min, and SpO₂ >95% on room air.

The patient was subsequently started on intravenous ceftriaxone and resumed oral antihypertensive medications (hydralazine and nifedipine), which gradually controlled her blood pressure. She was later transferred to another facility, where she completed a four-day course of intravenous piperacillin/tazobactam and was discharged fully recovered. No recurrence of confusion was noted during follow-up.

## Discussion

The unexpected improvement in this patient's GCS following ketamine administration aligns with emerging literature describing ketamine’s potential neuroactivating effects. This phenomenon has been documented in comparative studies evaluating ketamine versus other sedatives in terms of cognitive outcomes [9], as well as in experimental models of psychiatric and neurological illness [10].

One of the most clinically relevant examples of ketamine’s neurocognitive influence comes from its use in treatment-resistant major depressive disorder (MDD) [11]. Multiple randomized trials and systematic reviews have demonstrated that subanesthetic doses of ketamine can produce rapid and significant antidepressant effects-often within hours-suggesting a direct action on brain circuits involved in mood regulation, cognition, and consciousness [12]. This well-established role in MDD provides a biological and clinical rationale to explore ketamine’s influence on cortical arousal and recovery of consciousness in non-psychiatric contexts.

These findings challenge the traditional view of ketamine solely as a sedative or analgesic and support the hypothesis that ketamine may modulate thalamocortical connectivity, glutamatergic transmission, and cortical responsiveness. Further research is warranted to understand better ketamine’s role in patients presenting with acute confusion, delayed awakening, or impaired consciousness. Its use in critical care settings could potentially extend beyond sedation, facilitating ventilator weaning, early neurocognitive recovery, and even cerebral protection; however, these implications are speculative and based on emerging experimental evidence rather than established guideline recommendations.

This case is not merely an anecdote; it invites clinicians and researchers to explore ketamine's untapped therapeutic roles. It raises important clinical questions and may contribute to the development of future protocols targeting altered mental status and cognitive dysfunction. Ketamine continues to surprise, and its full potential is still being uncovered.

## Conclusions

This case describes a striking temporal association between low-dose ketamine and rapid resolution of acute confusion in an elderly patient. Although causality cannot be definitively established from a single report, the timing of neurological improvement following ketamine administration is striking. Emergency physicians should be aware of this possible neuroactivating phenomenon. Further targeted research is warranted to explore ketamine’s broader neurological benefits in emergency and critical care contexts.
